# Peripheral blood circular RNA circ-0008102 may serve as a novel clinical biomarker in beta-thalassemia patients

**DOI:** 10.1007/s00431-023-05398-y

**Published:** 2024-01-02

**Authors:** Meihuan Chen, Aixiang Lv, Siwen Zhang, Junhao Zheng, Na Lin, Liangpu Xu, Hailong Huang

**Affiliations:** 1 Medical Genetic Diagnosis and Therapy Center, Fujian Maternity and Child Health Hospital, College of Clinical Medicine for Obstetrics & Gynecology and Pediatrics, Fujian Medical University, Fujian Provincial Key Laboratory of Prenatal Diagnosis and Birth Defect, Fuzhou, 350001 China; 2 The School of Medical Technology and Engineering, Fujian Medical University, Fuzhou, 350001 China

**Keywords:** Circ-0008102, β-thal, Biomarker, Blood transfusion, HbF

## Abstract

Circular RNA circ-0008102 has previously been found dysregulated in β-thalassemia (β-thal) in circRNAs microarray (GSE196682 and GSE241141). Our study is aimed at identifying whether circ-0008102 could be a novel biomarker in β-thal. The peripheral blood of pediatric β-thal patients with (*n* = 39) or without (*n* = 20) blood transfusion and healthy controls (*n* = 30) was selected. qRT-PCR, ROC curve analysis, Spearman correlation analysis, and FISH were used to analyze clinical value of circ-0008102. qRT-PCR confirmed that circ-0008102 expression in pediatric β-thal patients without blood transfusion was significantly higher. ROC curves analysis showed that the AUC of circ-0008102 for differentiating patients without blood transfusion from patients with blood transfusion and healthy controls with an AUC of 0.733 and 0.711. Furthermore, circ-0008102 expression was positively correlated with the levels of RBC, HbF, β-globin, and γ-globin mRNA, but was negatively corrected with the levels of HbA and Cr. circ-0008102 was mainly located in the cytoplasm. circ-0008102 could induce the activation of γ-globin and negatively regulate the expression of the five highest-ranking candidate miRNAs (miR-372-3p, miR-329-5p, miR-198, miR-152-5p, and miR-627-3p) in K562 cells.

*Conclusion*: We demonstrate that peripheral blood upregulated circ-0008102 may serve as a novel clinical biomarker for pediatric β-thal without blood transfusion.

**Table:** 

**What is known:** *• CircRNAs are known to be involved in various human diseases, and several circRNAs are regarded as a class of promising blood-based biomarkers for detection of β-thal.* *• CircRNAs exert biological functions by epigenetic modification and gene expression regulation, and dysregulated circRNAs in β-thal might be involved in the induction of HbF in β-thal.*	
**What is new:** *• Peripheral blood circ-0008102 maybe serve as a novel clinical biomarker for detection of pediatric β-thal without blood transfusion.* *• Circ-0008102 participates in the pathogenesis of β-thal through regulating γ-globin expression, and negatively regulates the expression of miR-372-3p, miR-329-5p, miR-198, miR-152-5p and miR-627-3p.*

**Supplementary Information:**

The online version contains supplementary material available at 10.1007/s00431-023-05398-y.

## Background

β-Thalassemia (β-thal) is an autosomal recessive hereditary anemia characterized by reduced or absent β-globin chain synthesis, which is most widespread among Mediterranean countries, Central Asia, Middle East, India, South America, countries alongside the North coast of Africa, and Southern China. It has been assessed that approximately 68,000 children are born with various thalassemia syndromes each year worldwide [1]. According to clinical manifestations, there are three main forms of β-thal, including β-thal major (TM), β-thal intermedia (TI), and β-thal carrier (TC, also called thalassemia minor). Moreover, β-thal can be clinically classified as transfusion-dependent thalassemia (TDT) and non-transfusion-dependent thalassemia (NTDT) according to the severity of the phenotype, caused by a wide spectrum of mutations in a homozygous or compound heterozygous state. The β-globin gene, located on chromosome 11p15.5, is responsible for the coding of β-globin chain. Patients with TM or TI are chiefly homozygotes or compound heterozygotes for β^0^ or β^+^ mutation, and patients with TC are heterozygotes for β^0^ or β^+^ mutation. Although no treatment is needed for individuals who are diagnosed as TC, patients with TM are mainly treated by long-term blood transfusion and its high cost has brought a heavy economic burden to the family and society [2]. Currently, bone marrow or stem cell transplantation is a curable treatment for patients with TM, but it is difficult to popularize clinically due to the limitation of matching and marrow source [3]. Thus, an increased knowledge about the pathophysiology of β-thal may contribute to identify novel treatment methods for this disease.

As research progresses, more and more biomarkers associated with β-thal have been identified, including traditional proteins (alpha-hemoglobin pool, transferrin receptor 1, and heat shock protein 70) and non-coding RNAs (ncRNAs) [4, 5]. At present, studies on these biomarkers are still at the stage of expression analysis and the relevant functional mechanisms have not yet been elucidated. As a specific type of ncRNAs, circular RNAs (circRNAs) are commonly regarded to be derived from precursor mRNAs, with a covalently closed loop structure and non-poly-adenylated tail. Because of this unique structure, circRNAs are stable in the various body fluids and are not easily degraded by Ribonuclease R (RNase R). In recent years, with the development of high-throughput sequencing analysis and bioinformatic methods, thousands of circRNAs have been identified [6]. circRNAs are evolutionarily conserved, exhibit cell and tissue-type specific expression across species, and have shown great potential in diagnosis, therapy, and prognosis for a variety of human diseases, including hematological diseases [7, 8]. For example, circ-MYBL2, a circRNA from MYBL2 gene, is reported to facilitate the progression of acute myeloid leukemia by regulating fms-related receptor tyrosine kinase 3 (FLT3) translation through recruiting polypyrimidine tract binding protein 1 (PTBP1) [9]. circ-BUB1B_544aa, generated from BUB1 mitotic checkpoint serine/threonine kinase B (BUB1B) gene, has the potential to aggravate multiple myeloma malignancy through evoking chromosomal instability [10]. Besides, in myelodysplastic syndrome, peripheral blood circ-100352, circ-104056, and circ-102817 are valuable for diagnosis and prognosis and are linked with some important cancer-related functions and pathways [11]. Nowadays, increasing study has revealed that large amounts of circRNAs are abnormally expressed in β-thal and play important roles in epigenetic modification and gene expression regulation [12]. However, the roles and clinical value of many circRNAs in β-thal still remain to be explored.

Here, circ-0008102, also known as circ-100654, is derived from ligand-dependent nuclear receptor corepressor (LCOR) gene and is largely functionally unknown. A previous expression profile of circRNAs by microarray from the GSE196682 dataset has revealed that circ-0008102 expression is upregulated in the peripheral blood from β-thal carriers with high HbF. However, the circRNA microarray data (GSE241141) from our lab have shown that circ-0008102 level was downregulated in β-thal patients. These results suggested that circ-0008102 is dysregulated in β-thal. As a new circRNA, the role and mechanism of circ-0008102 in β-thal remain unknown. Our pre-experiments revealed that circ-0008102 expression in peripheral blood of pediatric β-thal patients was associated with HbF level. Therefore, whether increased circ-0008102 expression is involved in the development of β-thal is worth investigating. The present study is aimed at confirming the expression levels of circ-0008102 in large cohort of pediatric β-thal patients and healthy controls and analyzing its relationship with clinical characteristics. The receiver operating characteristic (ROC) curve analysis was performed to evaluate the diagnostic value of circ-0008102 for pediatric β-thal. Moreover, based on bioinformatics analysis, the circ-0008102/miRNA/mRNA interaction network was established to help us better understand its biological roles and underlying mechanisms in pediatric β-thal.

## Materials and methods

### Study participants

Fifty-nine pediatric β-thal patients (age 4–13 years, average age 8.12 ± 2.12 years) (20 cases without any blood transfusion for at least the past month prior to sample collection and 39 cases with blood transfusion history before the past month prior to sample collection) were prospective collected in Fujian Province and Guangxi Province, China, between January 2019 and December 2020. Thirty age- and gender-matched healthy controls (age 4–11 years, average age 7.27 ± 2.08 years) were obtained from Fujian Maternity and Child Health Hospital College of Clinical Medicine for Obstetrics and Gynecology and Pediatrics, Fujian Medical University. Including criteria: (1) β-thal patients have anemia symptoms and diagnosed with β-thal by genetic diagnosis; (2) healthy controls have no symptoms of anemia and no pathogenic mutation in the β-globin gene by genetic diagnosis. Exclusion criteria: (1) patients with other chronic and congenital diseases; (2) patients with acute and chronic lung infection; (3) patients with abnormal blood coagulation; (4) patients were under treatment with hydroxyurea; (5) patients with the three common deletional mutations (–SEA, -α3.7, and -α4.2) and three non-deletional mutations (αQSα, αCSα, and αWSα). All subjects had no genetic relationship. This study was approved by the Ethics Review Committee of Fujian Maternity and Child Health Hospital College of Clinical Medicine for Obstetrics and Gynecology and Pediatrics, Fujian Medical University (no: 073,2019) and was conducted in conformity to the Declaration of Helsinki. Written informed consent was obtained from all participants or their parents following a detailed description of the purpose of the study.

### Peripheral blood samples

The peripheral blood (5 ml each) was collected into PAXgene Blood RNA Tubes (Qiagen, Hilden, Germany) and was stored immediately at − 80 °C for further RNA extraction. After mixing by inversion of the tubes, approximately 2 ml peripheral blood samples anticoagulated with EDTA-K2 were collected for further hematological analysis. Hematological parameters, including red blood cells (RBC), hemoglobin (Hb), mean corpuscular volume (MCV), and mean corpuscular hemoglobin (MCH), were measured using a Sysmex XN-3000 automatic hematology analyzer (Sysmex Corporation; Shanghai, China). The hemoglobin components and levels, including hemoglobin A (HbA), hemoglobin A_2_ (HbA_2_), and fetal hemoglobin (HbF), were analyzed using an automated capillary S2 electrophoresis system version 6.2 (Sebia, Paris, France).

### Blood biochemical analysis

The biochemical parameters, including renal function ((blood urea nitrogen (BUN), creatinine (Cr), and uric acid (UA)), hepatic function ((total Protein (TP), albumin (ALB), alanine transaminase (ALT), aspartate transaminase (AST), gamma glutamyl-transferase (GGT), total bilirubin (TBIL), and direct bilirubin (DBIL)), and serum ferritin (SF), were determined using chemiluminescent microparticle immunoassay under an automatic biochemical analyzer (Abbott Diagnostics; Abbott Park, IL, USA).

### Molecular analysis for β-thal genotypes

Human genomic DNA (gDNA) was extracted from the peripheral blood samples using a genomic DNA isolation kit (Qiagen) following the manufacturer’s instruction. The DNA samples were quantified by a Bio Photometer MULTISKAN GO (Thermo Scientific, Waltham, MA, USA), and the concentrations greater than 100 ng/µL and the purity with 1.8–2.0 optical densities at 260/280 nm indicated good quality. The 17 common mutations of β-thal, including {codons (CD)41/42(-TCTT), IVS-II-654(C > T), − 28(A > G), CD71/72(+ A), CD17(A > T), HbE[β26(B8)Glu-Lys, GAG > AAG or CD26(G > A)], CD31(− C), CD27/28(+ C), CD43(G > T), − 32(C > A), − 29(A > G), − 30(T > C), CD14/15(+ G), Capt40 to t43(− AAAC), initiation CD(T > G), IVS-I-1(G > T), and IVS-I-5(G > T)}, were detected using reverse dot blot hybridization (RDBH) with a β-thal gene detection kit (Yishengtang Biological Products Co., Ltd, Shenzhen, China), following the manufacturer’s instruction.

### RNA extraction and reverse transcription

Total RNA from the peripheral blood samples was isolated using a PAXgene Blood RNA Kit (Qiagen) according to the manufacturer’s protocol. The purity of the RNA samples was assessed using a Bio Photometer MULTISKAN GO, and the OD260/280 ratio at 1.9 to 2.0 indicated good quality. The RNA integrity was determined by 1% formaldehyde-denatured gel electrophoresis. After calculating the concentration, RNA was frozen in − 80 °C refrigerator. RNA was reverse transcribed into cDNA using a SuperScript™ III Reverse Transcriptase (Invitrogen, Carlsbad, CA, USA) with random primers, according to the manufacturer’s protocol. The cDNA was preserved in − 20 °C for further use.

### Reverse transcription PCR (RT-PCR)

RT-PCR was carried out with cDNA and gDNA as templates to amplify circ-0008102 and LCOR mRNA using a PrimeScript™ RT-PCR kit, according to the manufacturer’s instruction (Takara Bio, Inc., Shiga, Japan). The procedures for RT-PCR were shown as follows: (1) denaturation at 98 °C for 10 min; (2) 35 step cycles of incubation at 98 °C for 10 s, 60 °C for 30 s, and 72 °C for 30 s; (3) a final extension at 72 °C for 5 min. The divergent primers were used to detect backsplice junction of circ-0008102, and convergent primers were applied to detect LCOR mRNA. Divergent primers: sense, 5′-GACGGTGTACTTGATCTGTCCA-3′ and antisense, 5′-GTTGGATCATTCGCTGCATGAT-3′; convergent primers: sense, 5′-GACGGACTTCGGAGTGGTGATG-3′ and antisense, 5′-GAGCCAGTGGAACTTTGAGTGATG-3′. After the reaction, 6 μL portion of each PCR product was analyzed by electrophoresis through a 2% agarose gel in 1 × Tris–borate-EDTA buffer at 15 V/cm for 50 min.

### Quantitative real-time PCR (qRT-PCR)

The cDNA of miRNAs was synthesized with a Mir-XTM First Strand Synthesis kit (Takara Bio, Inc.,), according to the manufacturer’s instructions. The StepOnePlus™ Real-Time PCR System (Applied Biosystems, Foster City, CA, USA) was adopted to perform qRT-PCR with a PrimeScript™ RT-PCR kit and Mir-X miRNA qRT-PCR TB Green® Kit (Takara Bio, Inc.,), according to the manufacturer’s instruction. The primers of circ-0008102, LCOR, β-globin, γ-globin, miR-372-3p, miR-329-5p, miR-198, miR-152-5p, and miR-627-3p were shown in Supplementary Table S1. The β-actin and U6 were used as the internal control for quantitative analysis. qRT-PCR was performed in triplicate for both target and internal control genes, and the negative control (non-cDNA) was run with every experimental plate to assess specificity and to rule out contamination. The relative fold-change of each target gene with respect to β-actin and U6 was calculated by the 2^−ΔΔCt^ method.

### RNAse R treatment

The RNAse R reagent (Epicentre Biotechnologies, Madison, WI, USA) was used to digest linear RNAs. Owing to circular structure, circRNAs could avoid RNase degradation. The total RNA was divided into 2 parts (2 μg each): one was for the digestion of 3 U/μg RNase R at 37 °C for 20 min; another was treated with equal amount of RNAse-free water under the same conditions. Subsequently, the RNA was reverse-transcribed into cDNA and qRT-PCR helped to analyze the detected LCOR mRNA and circ-0008102 following the above experimental methods.

### Sanger sequencing

Sanger sequencing was used to validate the back-splice junction sequences of circ-0008102. Briefly, the partial sequence fragments of circ-0008102 that contained the back-splice junction were amplified from cDNA using a PrimeSTAR® GXL Premix (Takara Bio, Inc.), according to the manufacturer’s recommendation. The amplification primers were as follows: sense, 5′-CCTGCCGAAAGCATCTCCAGT-3′ and antisense, 5′-TCGCTGCATGATCTCACGG-3′. The amplification primers were also used as primers for sequencing. Sanger sequencing was performed on an ABI 3730XL DNA analyzer (Applied Biosystems) and was analyzed by using a PC ChromasPro software version 2.1.5 (Applied Biosystems).

### Cell culture and cell transfection

Human K562 cells were purchased from the Cell Center of Shanghai Institutes for Biological Sciences (Shanghai, China). K562 cells were cultured in RPMI-1640 medium (Gibco, Grand Island, NY, USA), supplemented with 10% FBS (Gibco) and 1% penicillin–streptomycin (Sigma-Aldrich, Louis, MO, USA). All cells were maintained at 37 °C in a humidified atmosphere containing 5% CO_2_.

The circ-0008102 overexpressed vector, control vector, small-interfering RNA specifically targeting circ-0008102 (si-circ-0008102; sense: 5′-GAACGGGGACACUUAGUCGTT-3′, antisense: 5′-CGACUAAGUGUCCCCGUUCTT-3′), and si-control (sense: 5′-UUCUCCGAACGUGUCACGUTT-3′, antisense: 5′-ACGUGACACGUUCGGAGAATT-3′) were synthesized by GenePharma (Shanghai, China). The cell transfection of vectors and oligonucleotides was transfected into K562 cells using Zeta Life Advanced DNA RNA transfection reagent (Zeta Life, Menlo Park, CA, USA), according to the manufacturer’s instruction. The cells were harvested 24 h after transfection, and qRT-PCR was used to verify transfection efficiency.

### Fluorescence in situ hybridization (FISH)

The FISH kits of circ-0008102 and negative control were designed and purchased from GenePharma (Shanghai, China). Approximately 6 × 10^4^ K562 cells were seeded on cell climbing in 24-well plates overnight. Then, the cells were washed with PBS and fixed in 4% paraformaldehyde for 15 min at 37 °C and followed by permeabilized them for 15 min in 0.1% buffer A at 37 °C. After being washed with PBS, 2 × buffer C was added and maintained for 30 min at 37 °C, and then buffer E was added and rehydrated for 30 min at 73 °C. Subsequently, 1 μL of biotin-labeled circ-0008102-probe (5′-AGTGTCCCCGTTCCCTTGAGTACTG-3′) or negative control-probe (5′-TGCTTTGCACGGTAACGCCTGTTTT-3′), 1 μL of SA-Cy3, and 8 μL of PBS were added into the cells and incubated at 37 °C overnight by avoiding light. The next day, the cells were sequentially washed with 0.1% buffer F for 10 min at 37 °C, 2 × buffer C for 10 min (repeat 3 times) at 60 °C, and 2 × buffer C for 10 min (repeat 3 times) at 37 °C. Finally, the nucleus of cells was dyed in 4′,6′-diamidino-2-phenylindole (DAPI), and the subcellular localization of circ-0008102 was detected using a Leica TCS SP8 CARS Confocal Microscope (Leica, Wetzlar, Germany).

### Bioinformatics tools

The sequence of circ-0008102 was annotated and provided from circBase (http://www.circbase.org/). The circ-0008102 sponged miRNAs were predicted with starbase (http://starbase.sysu.edu.cn/), circBANK (http://www.circbank.cn/), and circinteractome (https://circinteractome.nia.nih.gov/). The target prediction of miRNAs was based on TargetScan (version 8.0; https://www.targetscan.org/vert_80/) and miRDB (version V6; http://mirdb.org/miRDB/). Cytoscape (version 3.8.2; http://www.cytoscape.org/) was applied to build the circ-0008102/miRNA/mRNA interaction network, and the mRNAs in the network were annotated using Gene Oncology (GO, https://geneontology.org/) and Kyoto Encyclopedia of Genes and Genomes (KEGG, https://www.kegg.jp/kegg/kegg1.html) pathway enrichment analysis [13–15].

### Statistical analysis

GraphPad Prism software (version 9.0; GraphPad Software Inc., San Diego, CA, USA) and SPSS software (version 25.0; SPSS, Chicago, IL, USA) were used for statistical analyses. Measurement data were expressed as the mean ± standard deviation (SD), and count data was expressed as case (%). The normality of the sample distributions was calculated by the Kolmogorov–Smirnoff test. The Mann–Whitney *U* test or independent *t*-test was used to assess the differences between the pediatric β-thal patients and healthy controls and the differences between pediatric β-thal patients with blood transfusion and pediatric β-thal patients without blood transfusion. The correlations between the expression level of circ-0008102 and hematological parameters, biochemical indicators, and β-globin and γ-globin mRNA expression were evaluated by Spearman correlation analysis. The ROC curve analysis and the area under curve (AUC) were carried on to detect circ-0008102 as biomarker for differentiating pediatric β-thal with or without blood transfusion. *P* values less than 0.05 were considered statistically significant.

## Results

### Comparison of baseline characteristics in pediatric β-thal patients and healthy controls

The genotypes of pediatric β-thal patients were shown in Supplementary Table S2, and a total of 19 genotypes were found in the pediatric β-thal patients (*n* = 59), and the pathogenic mutations of β-globin gene were mainly concentrated in the βCD41-42(-TCTT) (HBB:c.126_129delCTTT), β-28(A > G) (HBB:c.-78A > G), βIVS-II-654(C > T) (HBB:c.316-197C > T), βCD26(G > A) (HBB:c.79G > A), βCD71-72(+ A) (HBB:c.216_217insA), βCD27-28(+ C) (HBB:c.84_85insC), βInt(ATG > AGG) (HBB:c.2 T > G), βIVS-I-1(G > T) (HBB:c.92 + 1G > T), and βCD17(A > T) (HBB:c.52A > T) sites. No pathogenic mutation of β-globin gene was detected in the healthy controls (*n* = 30).

There was no statistical difference of age and sex distribution between pediatric β-thal patients and healthy controls. As expected, compared with healthy controls, pediatric β-thal patients displayed significantly decreased levels of hematological parameters, including the levels of RBC, Hb, MCH, and HbA (*P* < 0.001) (Table 1). But the levels of HbA_2_ (*P* = 0.008) and HbF (*P* < 0.001) in pediatric β-thal patients were significantly higher than that in healthy controls. In addition, pediatric β-thal patients showed abnormal biochemical indicators, including TBIL, DBIL, ALT, AST, and SF, and the levels of these parameters were markedly enhanced compared with that in healthy controls (*P* < 0.001); however, the levels of Cr and TP in pediatric β-thal patients were decreased compared with healthy controls (*P* < 0.01). There was no statistical difference in MCV, BUN, UA, ALB, and GGT between pediatric β-thal patients and healthy controls. These data confirmed that pediatric β-thal patients had phenotypes of anemia, abnormal liver function, and iron metabolism.

**Table 1:** Baseline characteristics of pediatric β-thal patients and healthy controls

Characteristics	Pediatric β-thal patients (*n* = 59)	Healthy controls (*n* = 30)	*P* values
Age (years)	8.12 ± 2.12	7.27 ± 2.08	0.083
Sex (male/female)	31/28	18/12	0.506
RBC (× 10^12^/L)	3.46 ± 0.63	4.64 ± 0.21	<0.001*
Hb (g/L)	91.39 ± 18.16	132.97 ± 5.68	<0.001*
MCV (fL)	80.16 ± 10.97	83.31 ± 2.20	0.124
MCH (pg)	26.33 ± 1.78	28.65 ± 0.85	<0.001*
HbA (%)	87.16 ± 11.62	96.74 ± 0.96	<0.001*
HbA_2_ (%)	3.79 ± 2.76	2.80 ± 0.17	0.008*
HbF (%)	9.05 ± 11.67	0.17 ± 0.34	<0.001*
BUN (mmol/L)	5.27 ± 2.00	4.79 ± 0.97	0.310
Cr (μmol/L)	30.77 ± 8.54	39.27 ± 6.07	<0.001*
UA (μmol/L)	288.98 ± 106.32	307.79 ± 72.06	0.478
TP (g/L)	68.20 ± 4.95	71.98 ± 4.75	0.004*
ALB (g/L)	44.22 ± 2.63	45.17 ± 1.85	0.177
TBIL (μmol/L)	29.69 ± 6.18	8.07 ± 2.42	<0.001*
DBIL (μmol/L)	9.59 ± 4.32	1.84 ± 0.56	<0.001*
ALT (U/L)	45.73 ± 40.00	13.87 ± 3.44	<0.001*
AST (U/L)	44.51 ± 28.78	25.49 ± 4.26	<0.001*
GGT (U/L)	15.97 ± 8.35	13.78 ± 2.43	0.284
SF (μg/L)	2873.47 ± 1657.40	48.30 ± 21.90	<0.001*

*RBC* red blood cells, *Hb* hemoglobin, *MCV* mean corpuscular volume, *MCH* mean corpuscular hemoglobin, *HbA* hemoglobin A, *HbA2* hemoglobin A_2_, *HbF* fetal hemoglobin, *BUN* blood urea nitrogen, *Cr* creatinine, *UA* uric acid, *TP* total protein, *ALB* albumin, *TBIL* total bilirubin, *DBIL* direct bilirubin, *ALT* alanine transaminase, *AST* aspartate transaminase, *GGT* gamma glutamyl-transferase, *SF* serum ferritin

*P < 0.05

Moreover, based on blood transfusion or not, the pediatric β-thal patients were further categorized into two groups: without blood transfusion group (*n* = 20) and blood transfusion group (*n* = 39). The hematological parameters and biochemical indicators of the two groups were compared and shown in Supplementary Table S3. Compared with that in patients without blood transfusion, patients with blood transfusion showed higher levels of Hb, MCV, MCH, and HbA and lower level of HbF (*P* < 0.05). However, no significant difference was observed in age, sex distribution, RBC, HbA_2_, BUN, Cr, UA, TP, ALB, TBIL, DBIL, ALT, AST, GGT, and SF levels in the two groups (*P* > 0.05). These data demonstrated that blood transfusion could improve anemia indicators and was an effective treatment option for β-thal.

### Identification and characterization of circ-0008102

**Fig. 1:**
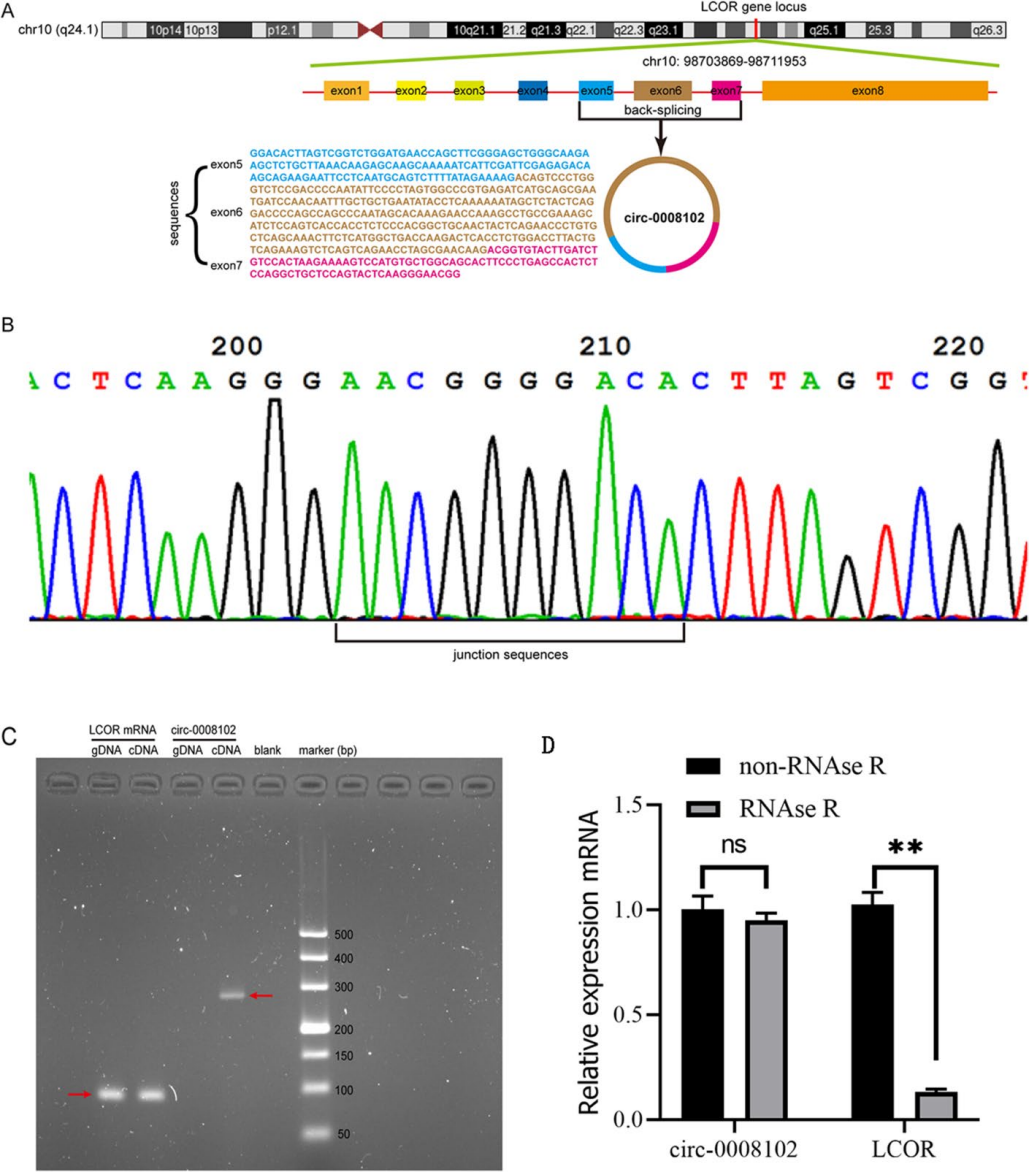
Identification of the circular structure of circ-0008102. **A** The genomic locus and back-spliced junction of circ-0008102 were shown in schematic diagram. **B** The junction sequences of circ-0008102 were validated by Sanger sequencing. **C** RT-PCR analysis of circ-0008102 and LCOR mRNA expression in cDNA and gDNA from pediatric β-thal using divergent and convergent primers, respectively. **D** qRT-PCR analysis of circ-0008102 and LCOR mRNA levels in K562 cells under treatment with RNAse R. RT-PCR, reverse transcription PCR; LCOR, ligand dependent nuclear receptor corepressor; gDNA, genomic DNA; qRT-PCR, quantitative real-time PCR; RNase R, Ribonuclease R. Error bars represented the means ± SD. **P < 0.01, ^ns^*P* > 0.05

Based on the annotation of circBase, circ-0008102 with a length of 515 nt was produced by back-splicing from exons 5, 6, and 7 of the LCOR gene locus (NM_032440), which was located at human chromosome 10: 98,703,869–98711953 position (strand +, GRCh37/hg19) (Fig. 1A). The junction sequence of circ-0008102 was validated by Sanger sequencing (Fig. 1B). Subsequently, to verify that circ-0008102 was generated from the back-splicing instead of trans-splicing or genomic rearrangements, the divergent and convergent primers were designed to amplify circ-0008102 and LCOR mRNA, respectively. RT-PCR revealed that both cDNA and gDNA could amplify LCOR mRNA using the convergent primers, whereas only cDNA could amplify circ-0008102 using the divergent primers (Fig. 1C). Additionally, qRT-PCR showed that circ-0008102 was more stable than LCOR mRNA in K562 cells under treatment with RNAse R (Fig. 1D, P < 0.01). To summarize, circ-0008102 was confirmed to be a circular RNA.

### Validation of upregulated circ-0008102 expression in pediatric β-thal patients without blood transfusion

**Fig. 2:**
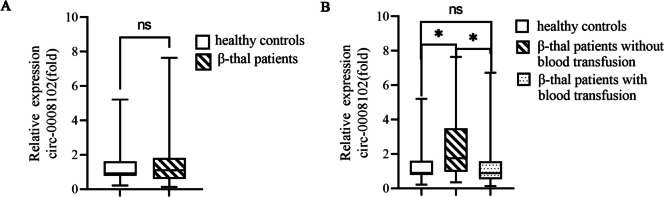
The upregulated expression of circ-0008102 in pediatric β-thal patients without blood transfusion. **A** qRT-PCR analysis of the expression levels of circ-0008102 (normalized to β-actin) in peripheral blood of pediatric healthy controls and pediatric β-thal patients. **B** The circ-0008102 expression was upregulated in pediatric β-thal patients without blood transfusion relative to pediatric β-thal patients with blood transfusion and healthy controls. Data were presented as the means ± SD. *P < 0.05, ^ns^*P* > 0.05

We explored the expression levels of circ-0008102 in peripheral blood of large cohort of subjects, and results showed that there was no statistical difference of circ-0008102 expression between pediatric β-thal patients and healthy controls (Fig. 2A, *P* > 0.05). Surprisingly, the expression levels of circ-0008102 in peripheral blood of pediatric β-thal patients without blood transfusion were significantly higher than that in pediatric β-thal patients with blood transfusion and healthy controls (Fig. 2B, *P* < 0.05). These data demonstrated that circ-0008102 expression was upregulated in pediatric β-thal patients without blood transfusion.

### Evaluation of circ-0008102 in peripheral blood as a novel diagnostic biomarker for pediatric β-thal patients without blood transfusion

**Fig. 3:**
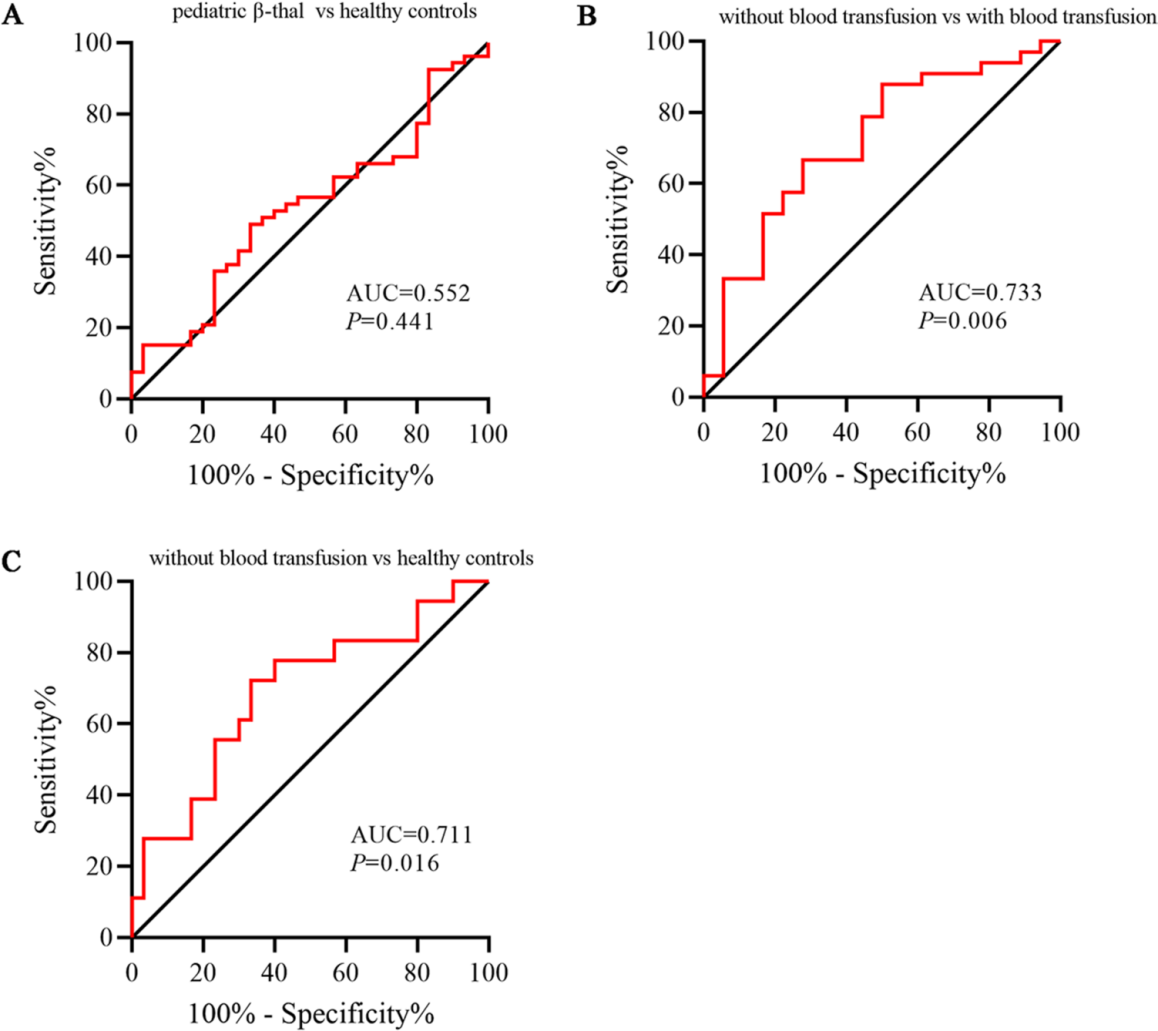
Evaluation of circ-0008102 as a novel diagnostic biomarker for pediatric β-thal patients without blood transfusion. ROC curves analysis of peripheral blood circ-0008102 for discriminating **A** pediatric β-thal patients from healthy controls, **B** pediatric β-thal patients without blood transfusion from pediatric β-thal patients with blood transfusion, and **C** pediatric β-thal patients without blood transfusion from healthy controls. ROC, receiver operating characteristic; AUC, the area under curve

We then evaluated the diagnostic ability of circ-0008102 to distinguish pediatric β-thal patients from healthy controls through ROC curves analysis. Figure 3A showed that the AUC value was 0.552, and 95% confidence interval (CI) was 0.424–0.679, and the cut-off value was 1.11, with sensitivity was 0.491 and specificity was 0.690 (Fig. 3A, *P* = 0.441), hinting that circ-0008102 had low clinical diagnostic value for the diagnosis of pediatric β-thal. As shown in Fig. 3B, peripheral blood circ-0008102 expression discriminated pediatric β-thal patients without blood transfusion from pediatric β-thal patients with blood transfusion with an AUC of 0.733 (95% CI 0.590–0.877, sensitivity 0.722, and specificity 0.686) (*P* = 0.006). Similarly, the AUC of circ-0008102 for differentiating pediatric β-thal patients without blood transfusion from healthy controls was 0.711 (95% CI 0.554–0.868), with 0.722 sensitivity and 0.690 specificity (Fig. 3C, *P* = 0.016). These data demonstrated peripheral blood circ-0008102 might be a novel clinical biomarker in pediatric β-thal without blood transfusion.

### Analysis of correlation between circ-0008102 expression and clinical characteristics in pediatric β-thal patients without blood transfusion

As shown in Supplementary Table S4, the association between circ-0008102 expression and hematological parameters in pediatric β-thal patients without blood transfusion showed that the expression level of circ-0008102 was negatively correlated with the level of HbA (*r* = − 0.494, *P* = 0.037), but positively correlated with the levels of RBC (*r* = 0.521, *P* = 0.026) and HbF level (*r* = 0.480, *P* = 0.044). However, no association was found between circ-0008102 expression and Hb, MCV, MCH, and HbA_2_ (all *P* > 0.05)_._ Similarly, Spearman correlation analysis revealed that the expression level of circ-0008102 was negatively correlated with the level of Cr (*r* = − 0.726, *P* = 0.018) in pediatric β-thal patients without blood transfusion. However, no association was found in BUN, UA, TP, ALB, TBIL, DBIL, ALT, AST, GGT, and SF (all *P* > 0.05)_._ Together, these data suggested that upregulated circ-0008102 expression was associated with the pathogenesis of pediatric β-thal without blood transfusion.

### Analysis of β-globin and γ-globin expression in pediatric β-thal patients without blood transfusion and its association with circ-0008102 expression

**Fig. 4:**
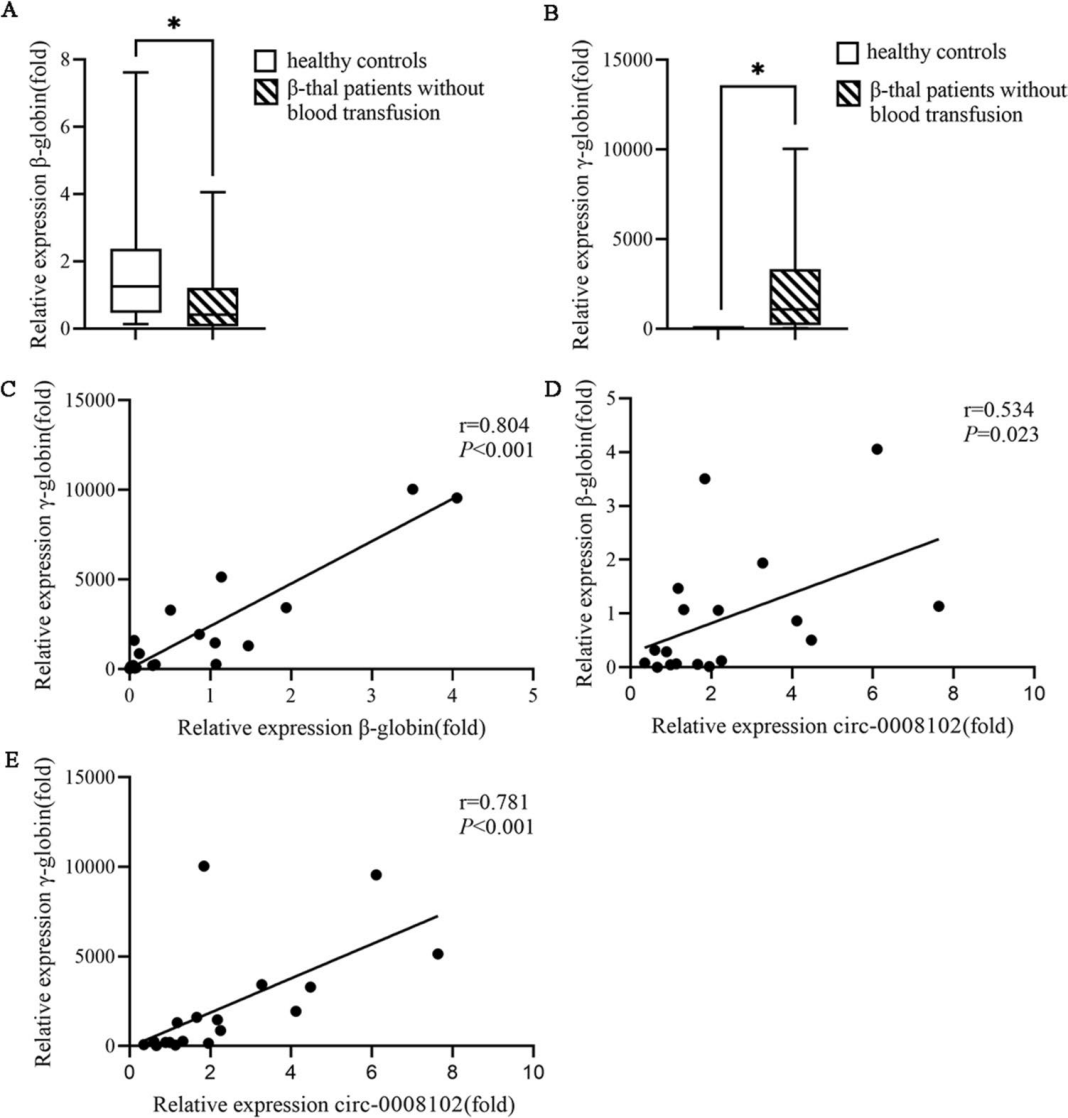
The association between circ-0008102 expression and β-globin and γ-globin levels in pediatric β-thal patients without blood transfusion. **A** qRT-PCR showed that the expression levels of β-globin mRNA were downregulated in pediatric β-thal patients. **B** qRT-PCR revealed that the expression levels of γ-globin mRNA were upregulated in pediatric β-thal patients. **C** Spearman correlation coefficient between the expression of β-globin and γ-globin mRNA in pediatric β-thal patients. **D** circ-0008102 expression was positively correlated with β-globin mRNA level in pediatric β-thal patients. **E** circ-0008102 level was positively correlated with γ-globin mRNA expression in pediatric β-thal patients. Error bars represented the means ± SD. *P < 0.05

Compared with healthy controls, the mRNA expression levels of β-globin in the peripheral blood from pediatric β-thal patients without blood transfusion were significantly downregulated with 0.411 fold changes (Fig. 4A, *P* < 0.05); however, the mRNA expression levels of γ-globin were significantly upregulated with 1087.09 fold changes (Fig. 4B, *P* < 0.05). A positive correlation between the mRNA expression levels of β-globin and γ-globin was shown in Fig. 4C (*r* = 0.804, *P* < 0.001). Furthermore, in pediatric β-thal patients without blood transfusion, circ-0008102 expression was positively correlated with the mRNA expression levels of β-globin (*r* = 0.534) and γ-globin (*r* = 0.781) (Fig. 4D, E, *P* < 0.05). Moreover, the mRNA expression levels of γ-globin were regulated when circ-0008102 overexpression or knockdown (Supplementary Fig. S1,* P* < 0.05). All data stated that circ-0008102 might be involved in the regulation of γ-globin expression in pediatric β-thal without blood transfusion.

### Construction of circ-0008102/miRNA/mRNA interaction network

**Fig. 5:**
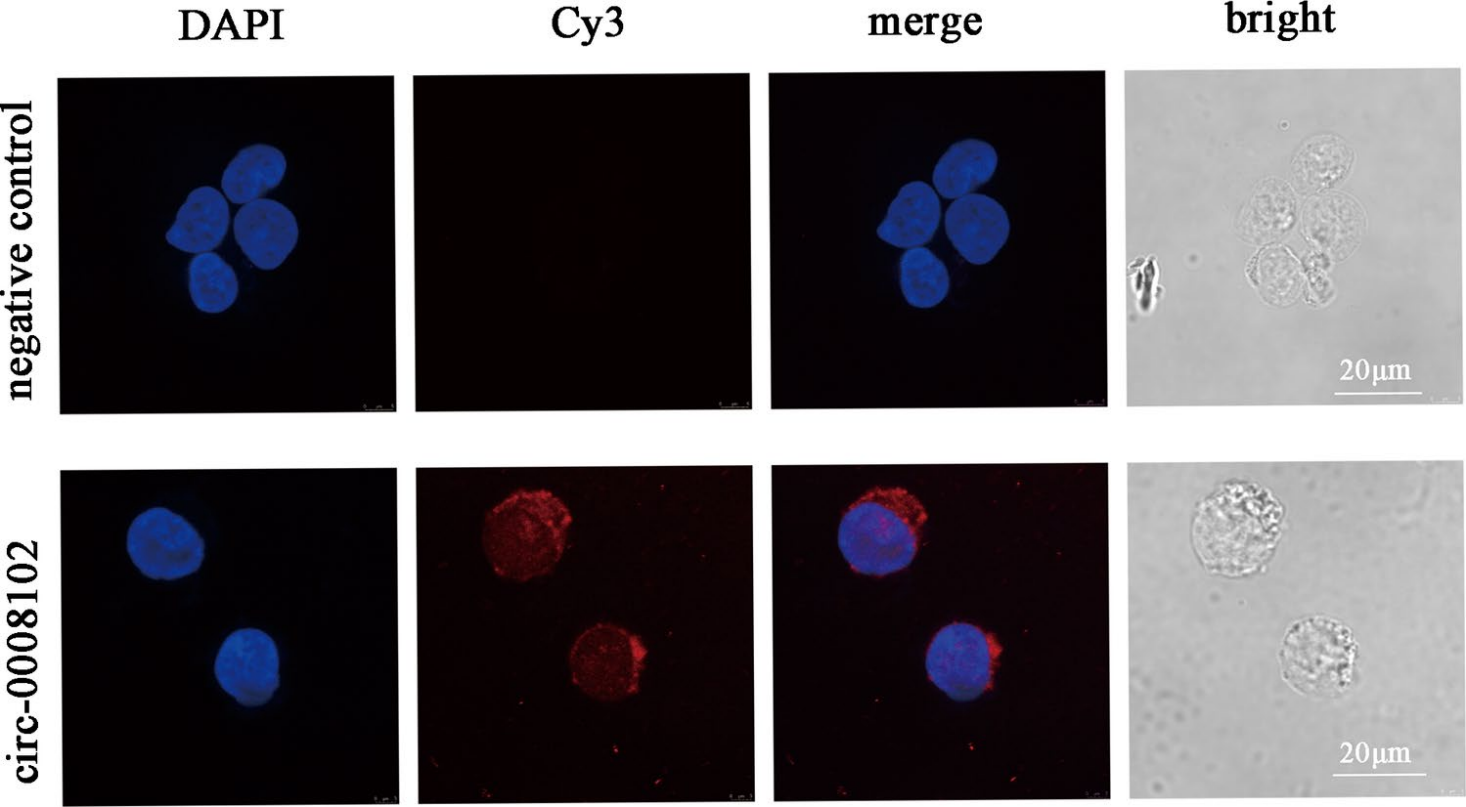
The subcellular localization of circ-0008102. Fluorescence in situ hybridization (FISH) found that circ-0008102 was mainly located in the cytoplasm of K562 cells, scale bar = 20 μm

The results of FISH showed that circ-0008102 was mainly located in the cytoplasm of K562 cells (Fig. 5), suggesting circ-0008102 could function as a miRNA sponge. The molecular interactions between circ-0008102 and its five highest-ranking candidate miRNAs regulated by circ-0008102 were displayed in Supplementary Fig. S2, including miR-372-3p, miR-329-5p, miR-198, miR-152-5p, and miR-627-3p. Moreover, the expression levels of the five miRNAs were detected in K562 cells after overexpression and knockdown of circ-0008102. We found that overexpression of circ-0008102 decreased miR-372-3p, miR-329-5p, miR-198, miR-152-5p, and miR-627-3p expression (Supplementary Fig. S3A,* P* < 0.001). Knockdown of circ-0008102 increased miR-372-3p, miR-329-5p, miR-198, miR-152-5p, and miR-627-3p expression in K562 cells (Supplementary Fig. S3B,* P* < 0.05). These data confirmed that miR-372-3p, miR-329-5p, miR-198, miR-152-5p, and miR-627-3p could be regulated by circ-0008102.

In addition, the target genes of those five miRNAs were predicted by TargetScan 8.0 and miRDB V6 databases, 651 mRNAs were found, and seventeen of them were targeted by more than one miRNAs, such as ryanodine receptor 2 (RYR2), VHL binding protein 1 (VBP1), leukocyte immunoglobulin-like receptor B5 (LILRB5), FYVE and coiled-coil domain autophagy adaptor 1 (FYCO1), ferritin mitochondrial (FTMT), and sterile alpha motif domain containing 1 (SAMD1). We further constructed the circ-0008102/miRNA/mRNA network focused on the five miRNAs and 651 mRNAs (Supplementary Fig. S4).

### GO and KEGG enrichment analyses of circ-0008102/miRNA/mRNA network

The top 10 significantly enriched GO terms in the biological process (BP), cellular components (CC), and molecular function (MF) were shown in Supplementary Fig. S5A-C. In terms of BP, the top three enriched items were “regulation of DNA-templated transcription,” “regulation of nucleic acid-templated transcription,” and “regulation of RNA biosynthetic process.” In terms of CC, the top three enriched items were “transcription regulator complex,” “chromation,” and “chromosome.” In terms of MF, “sequence-specific DNA binding,” “transcriptional cis-regulatory region binding,” and “transcription regulatory region nucleic acid binding” were enriched for the first three places. Besides, the top 10 significantly enriched pathways of KEGG enrichment analysis were shown in Supplementary Fig. S5D. Among these pathways, “TGF-beta signaling pathway,” “stem cells pluripotency,” “Th17 cell differentiation,” and “cellular senescence” were closely correlated with the pathogenesis of pediatric β-thal without blood transfusion.

## Discussion

With the deepening of the research on the underlying molecular basis of β-thal, its clinical treatment methods have been updated and gradually improved, and more and more biomarkers related to this disease have been reported, including erythrocyte transferrin receptor 1, alpha-hemoglobin stabilizer protein, heat shock protein 70, and CD35 and CD55 [16–18]. In recent years, ncRNAs, including miRNAs and long non-coding RNAs (lncRNAs), are regarded as a class of promising blood-based biomarkers for detection of human diseases including β-thal [19]. For example, Leecharoenkiat et al. reported that plasma upregulated miR-451 expression was a novel hemolytic marker in β^0^-thal/HbE patients and was associated with the degree of hemolysis and promoted erythropoiesis [20]. Kuno et al. demonstrated that elevated miR-125b expression from activated phagocytic monocytes was a genetic modifier related to anemia severity in β-thal patients [21]. El-Khazragy et al. suggested that plasma-dysregulated miR-let-7d, miR-122, and miR-200 served as novel and non-invasive predictor biomarkers for cellular damage under condition of tissue iron excess in transfusion-dependent β-thal patients [22]. Recently, a cross-sectional study concluded that circulating MALAT1 (metastasis-associated lung adenocarcinoma transcript 1) and MIAT (myocardial infarction-associated transcript) were promising predictors for blood transfusion status in β-thal patients [23]. Nevertheless, the potential biomarkers of β-thal remain largely unknown.

Accumulated evidence has suggested that the expression of circRNAs is tissue- and cell type-specific and specifically regulates disease progression [7]. Compared with proteins, miRNAs, lncRNAs, and circRNAs are ideal candidate biomarkers in human diseases, as they are abundant and stable in body fluids, including peripheral blood, serum, cerebrospinal fluid, urine, gastric fluid, and joint fluid [8]. Therefore, it is of great significance to identify circRNA-related biomarkers in β-thal. In this study, we found upregulated circ-0008102 expression was associated with the pathogenesis of pediatric β-thal and firstly demonstrated that circ-0008102 might be an effective biomarker for detection of pediatric β-thal without blood transfusion.

LCOR, as a master transcription factor, is involved in converting hemogenic endothelium into hematopoietic stem and progenitor cells [24]. It is also reported that LCOR negatively regulates early adipogenesis by inhibiting C/EBPβ transcriptional activity [25]. circ-0008102 is derived from LCOR gene locus, which is an exonic circRNA with 515 nt in length. Recently, dysregulated circRNAs have received more attention as diagnostic or predictive biomarkers for malignant tumors, nervous system diseases, cardiovascular disease, and endocrine diseases [26, 27]. However, the role of circRNAs in β-thal is poorly known. Herein, 59 pediatric β-thal patients were collected and their hematological parameters and biochemical indicators were consistent with the phenotypes of TM or TI. Encouragingly, we found that the expression levels of circ-0008102 in peripheral blood from pediatric β-thal patients without blood transfusion were significantly higher than that in pediatric β-thal patients with blood transfusion and healthy controls. The data were consistent with upregulated circ-0008102 expression in a circRNA microarray profile of GSE196682 dataset (adult β-thal patients vs. healthy controls), indicating high credibility of our data. Furthermore, we found circ-0008102 expression was positively correlated with the levels of RBC and HbF, but negatively corrected with the levels of HbA and Cr. Blood transfusion affected the expression of circ-0008102 in pediatric β-thal patients probably for the following reasons: (1) due to the low expression of circ-0008102 in healthy controls, blood transfusion led to a decrease in the expression of circ-0008102 in the peripheral blood of pediatric β-thal patients; (2) due to the positive correlation between circ-0008102 and HbF, HbF decreased after blood transfusion, which caused a decrease in circ-0008102 expression as a result of feedback regulation; (3) due to the negative correlation between circ-0008102 and HbA, an increase in HbA after blood transfusion led to a decrease in circ-0008102. All these strong unraveled upregulated circ-0008102 expression was associated with the pathogenesis of pediatric β-thal without blood transfusion. Therefore, the detailed biological role of circ-0008102 in pediatric β-thal deserved further study.

circRNAs display good diagnostic performance, not only because of their high specificity and sensitivity but also because of their great stability in peripheral blood [28]. Currently, the predictive role of circRNAs in β-thal remains to be blank. To our knowledge, it is the first time to identify the diagnostic power of circ-0008102 in pediatric β-thal patients in our study. The peripheral blood circ-0008102 revealed a significant diagnostic value on pediatric β-thal patients without blood transfusion when discriminated with pediatric β-thal patients with blood transfusion and healthy controls (AUC 0.733 and 0.711, respectively). These results suggested that circ-0008102 had potential as a novel biomarker in the detection of pediatric β-thal without blood transfusion. Without doubt, future multi-center, large-scale study is needed to verify the diagnostic value of circ-0008102 in pediatric β-thal.

Epigenetic factors can influence the severity of β-thal, leading to differing presentations despite similar underlying mutations. Increasing studies suggest that reactivating of HbF is an effective therapeutic target for β-thal [29, 30]. In β-thal patients, the γ-globin gene is activated to elevate the expression of HbF, resulting in reduced hemolysis and relieved the symptoms of anemia. The regulatory roles of ncRNAs on γ-globin expression have gradually been identified, such as miRNAs: miR-29b/miR-96/miR-326/miR-2355-5p [31–34] and lncRNAs: hemoglobin subunit beta pseudogene 1 (HBBP1)/HMI-LNCRNA/BGLT3-lncRNA [35–37]. In our study, we found an increased expression of γ-globin mRNA in pediatric β-thal patients, and γ-globin mRNA expression level had a positive correlation with β-globin mRNA expression, confirming the activated γ-globin gene in β-thal. Moreover, we found circ-0008102 expression was positively correlated with the mRNA levels of γ-globin and β-globin, indicating circ-0008102 might be involved in the regulation of γ-globin expression. In addition, we found overexpression of circ-0008102 increased γ-globin expression in K562 cells, while knockdown of circ-0008102 decreased γ-globin expression, suggesting that circ-0008102 could induce the activation of γ-globin in pediatric β-thal.

Increasing evidence showed that exonic circRNAs served as sponges of miRNAs to regulate the expression of target mRNAs [38]. We also found circ-0008102 was mainly located in cytoplasm of K562 cells by FISH assay, confirming circ-0008102 functioned as miRNA sponges. To better understand the regulatory mechanism of circ-0008102 in this study, we constructed the circ-0008102/miRNA/mRNA network based on bioinformatics analysis and found circ-0008102 might act as ceRNAs to capture miR-372-3p, miR-329-5p, miR-198, miR-152-5p, and miR-627-3p and subsequently regulated the 651 mRNA expression. Previous studies had demonstrated that the five miRNAs could be detected in peripheral blood [39–43], suggesting that they might be aberrantly expressed in pediatric β-thal. The negative regulatory effect of circ-0008102 on the five miRNAs was verified in K562 cells by overexpression and knockdown of circ-0008102, confirming circ-0008102/miRNA/mRNA network. The GO and pathway enrichment analyses indicated that these mRNAs were involved in DNA binding and transcription regulatory region binding and were associated with Th17 cell differentiation and stem cell pluripotency signaling pathways. All of these data indicated that circ-0008102 might be an effective biomarker for pediatric β-thal without blood transfusion.

The shortcomings of this study: (1) the expression levels of miR-372-3p, miR-329-5p, miR-198, miR-152-5p, and miR-627-3p in pediatric β-thal and their correlation with circ-0008102 expression remain unknown. (2) The expression profiles of circ-0008102/miRNA/mRNA network in public database of β-thal remain unknown. (3) The specific regulatory sites of circ-0008102 for the five miRNAs need to be experimentally verified. (4) The relationship between circ-0008102 expression and prognosis in pediatric β-thal remains unclear. (5) In addition to regulating the activation of γ-globin, the role of circ-0008102 in the progression of pediatric β-thal remains largely unknown. Future research will use in vitro and in vivo experiments to address these issues.

In conclusion, our data provide preliminary evidence that the expression level of circ-0008102 is upregulated in peripheral blood of pediatric β-thal without blood transfusion, and circ-0008102 may serve as a novel clinical biomarker for detection of pediatric β-thal without blood transfusion. circ-0008102 participates in the pathogenesis of β-thal through regulating γ-globin expression, which needs to be investigated further.

### Supplementary Information

Below is the link to the electronic supplementary material.

Supplementary file1 (TIF 5258 KB)

Supplementary file2 (TIF 2021 KB)

Supplementary file3 (TIF 3610 KB)

Supplementary file4 (TIF 3397 KB)

Supplementary file5 (TIF 808 KB)

Supplementary file6 (DOCX 14 KB)

Supplementary file7 (DOCX 20 KB)

Supplementary file8 (DOCX 17 KB)

Supplementary file9 (DOCX 15 KB)

## Data Availability

The datasets generated and/or analyzed during the current study are available in the GenBank repository (https://www.ncbi.nlm.nih.gov/genbank/; accession number: OR264515) and GEO DataSets (https://www.ncbi.nlm.nih.gov/gds/; accession number: GSE196682 and GSE241141).

## References

[1] Kattamis A, Forni GL, Aydinok Y, Viprakasit V (2020) Changing patterns in the epidemiology of beta-thalassemia. Eur J Haematol 105:692–703. 10.1111/ejh.13512 10.1111/ejh.13512 PMC7692954 32886826

[2] Betts M et al (2020) Systematic literature review of the burden of disease and treatment for transfusion-dependent beta-thalassemia. Clin Ther 42:322–337:e322. 10.1016/j.clinthera.2019.12.003 10.1016/j.clinthera.2019.12.003 31882227

[3] Ali S et al (2021) Current status of beta-thalassemia and its treatment strategies. Mol Genet Genomic Med 9:e1788. 10.1002/mgg3.1788 10.1002/mgg3.1788 PMC8683628 34738740

[4] Vasseur C et al (2017) Red blood cells free alpha-haemoglobin pool: a biomarker to monitor the beta-thalassemia intermedia variability. The ALPHAPOOL study. Br J Haematol 179:142–153. 10.1111/bjh.14800 10.1111/bjh.14800 28643346

[5] Wang F, Ling L, Yu D (2021) MicroRNAs in beta-thalassemia. Am J Med Sci 362:5–12. 10.1016/j.amjms.2021.02.011 10.1016/j.amjms.2021.02.011 33600783

[6] Kristensen LS et al (2019) The biogenesis, biology and characterization of circular RNAs. Nat Rev Genet 20:675–691. 10.1038/s41576-019-0158-7 10.1038/s41576-019-0158-7 31395983

[7] Zhou X, Zhan L, Huang K, Wang X (2020) The functions and clinical significance of circRNAs in hematological malignancies. J Hematol Oncol 13:138. 10.1186/s13045-020-00976-1 10.1186/s13045-020-00976-1 PMC7568356 33069241

[8] Zhang Z, Yang T, Xiao J (2018) Circular RNAs: promising biomarkers for human diseases. EBioMedicine 34:267–274. 10.1016/j.ebiom.2018.07.036 10.1016/j.ebiom.2018.07.036 PMC6116471 30078734

[9] Sun YM et al (2019) circMYBL2, a circRNA from MYBL2, regulates FLT3 translation by recruiting PTBP1 to promote FLT3-ITD AML progression. Blood 134:1533–1546. 10.1182/blood.2019000802 10.1182/blood.2019000802 PMC6839953 31387917

[10] Tang X et al (2021) BUB1B and circBUB1B_544aa aggravate multiple myeloma malignancy through evoking chromosomal instability. Signal Transduct Target Ther 6:361. 10.1038/s41392-021-00746-6 10.1038/s41392-021-00746-6 PMC8497505 34620840

[11] Wu WL, Li S, Zhao GJ, Li NY, Wang XQ (2021) Identification of circular RNAs as novel biomarkers and potentially functional competing endogenous RNA network for myelodysplastic syndrome patients. CancerSci 112:1888–1898. 10.1111/cas.14843 10.1111/cas.14843 PMC8088940 33560542

[12] Yang, F et al (2022) Analysis of circRNAs and circRNA-associated competing endogenous RNA networks in beta-thalassemia. Sci Rep 12:8071. 10.1038/s41598-022-12002-0 10.1038/s41598-022-12002-0 PMC9110710 35577924

[13] Kanehisa M, Goto S (2000) KEGG: Kyoto encyclopedia of genes and genomes. Nucleic Acids Res 28:27–30. 10.1093/nar/28.1.27 10.1093/nar/28.1.27 PMC102409 10592173

[14] Kanehisa M (2019) Toward understanding the origin and evolution of cellular organisms. Protein Sci 28:1947–1951. 10.1002/pro.3715 10.1002/pro.3715 PMC6798127 31441146

[15] Kanehisa M, Furumichi M, Sato Y, Kawashima M, Ishiguro-Watanabe M (2023) KEGG for taxonomy-based analysis of pathways and genomes. Nucleic Acids Res 51:D587–D592. 10.1093/nar/gkac963 10.1093/nar/gkac963 PMC9825424 36300620

[16] Ricchi P et al (2017) Soluble form of transferrin receptor-1 level is associated with the age at first diagnosis and the risk of therapeutic intervention and iron overloading in patients with non-transfusion-dependent thalassemia. Ann Hematol 96:1541–1546. 10.1007/s00277-017-3057-z 10.1007/s00277-017-3057-z 28707012

[17] Arlet JB et al (2014) HSP70 sequestration by free alpha-globin promotes ineffective erythropoiesis in beta-thalassaemia. Nature 514:242–246. 10.1038/nature13614 10.1038/nature13614 25156257

[18] Kurtogllu AU, Koctekin B, Kurtoglu E, Yildiz M, Bozkurt S (2017) Expression of CD55, CD59, and CD35 on red blood cells of beta-thalassaemia patients. Cent Eur J Immunol 42:78–84. 10.5114/ceji.2017.67321 10.5114/ceji.2017.67321 PMC5470617 28680334

[19] Rahaman M et al (2022) Exploring the crosstalk between long non-coding RNAs and microRNAs to unravel potential prognostic and therapeutic biomarkers in beta-thalassemia. Mol Biol Rep 49:7057–7068. 10.1007/s11033-022-07629-1 10.1007/s11033-022-07629-1 35717472

[20] Leecharoenkiat K et al (2017) Plasma microRNA-451 as a novel hemolytic marker for beta0-thalassemia/HbE disease. Mol Med Rep 15:2495–2502. 10.3892/mmr.2017.6326 10.3892/mmr.2017.6326 PMC5428399 28447765

[21] Kuno S, Penglong T, Srinoun K (2019) Anemia severity in beta-thalassemia correlates with elevated levels of microRNA-125b in activated phagocytic monocytes. Hemoglobin 43:155–161. 10.1080/03630269.2019.1628043 10.1080/03630269.2019.1628043 31379233

[22] El-Khazragy N et al (2021) Circulating miRNAs and tissue iron overload in transfusion-dependent beta-thalassemia major: novel predictors and follow-up guide. Ann Hematol 100:2909–2917.10.1007/s00277-021-04639-0 10.1007/s00277-021-04639-0 34432101

[23] Fakhr-Eldeen A, Toraih EA, Fawzy MS (2019) Long non-coding RNAs MALAT1, MIAT and ANRIL gene expression profiles in beta-thalassemia patients: a cross-sectional analysis. Hematology 24:308–317. 10.1080/16078454.2019.1570616 10.1080/16078454.2019.1570616 30665334

[24] Sugimura R et al (2017) Haematopoietic stem and progenitor cells from human pluripotent stem cells. Nature 545:432–438.10.1038/nature22370 10.1038/nature22370 PMC5872146 28514439

[25] Cao H et al (2017) Ligand-dependent corepressor (LCoR) represses the transcription factor C/EBPbeta during early adipocyte differentiation. J Biol Chem 292:18973–18987. 10.1074/jbc.M117.793984 10.1074/jbc.M117.793984 PMC5704479 28972158

[26] Zaiou M (2020) circRNAs signature as potential diagnostic and prognostic biomarker for diabetes mellitus and related cardiovascular complications. Cells 9:659. 10.3390/cells9030659 10.3390/cells9030659 PMC7140626 32182790

[27] Ma Y, Liu Y, Jiang Z (2020) CircRNAs: a new perspective of biomarkers in the nervous system. Biomed Pharmacother 128:110251. 10.1016/j.biopha.2020.110251 10.1016/j.biopha.2020.110251 32480219

[28] Haque S et al (2020) circRNAs expressed in human peripheral blood are associated with human aging phenotypes, cellular senescence and mouse lifespan. Geroscience 42:183–199. 10.1007/s11357-019-00120-z 10.1007/s11357-019-00120-z PMC7031184 31811527

[29] Gong Y et al (2021) A natural DNMT1 mutation elevates the fetal hemoglobin level via epigenetic derepression of the gamma-globin gene in beta-thalassemia. Blood 137:1652–1657. 10.1182/blood.2020006425 10.1182/blood.2020006425 33227819

[30] Zakaria NA et al (2021) Epigenetic insights and potential modifiers as therapeutic targets in beta-thalassemia. Biomolecules 11:755. 10.3390/biom11050755 10.3390/biom11050755 PMC8158146 34070036

[31] Starlard-Davenport A, Smith A, Vu L, Li B, Pace BS (2019) MIR29B mediates epigenetic mechanisms of HBG gene activation. Br J Haematol 186:91–100. 10.1111/bjh.15870 10.1111/bjh.15870 PMC6589104 30891745

[32] Azzouzi I et al (2011) MicroRNA-96 directly inhibits gamma-globin expression in human erythropoiesis. PLoS One 6:e22838. 10.1371/journal.pone.0022838 10.1371/journal.pone.0022838 PMC3145767 21829531

[33] Li Y et al (2018) miR-326 regulates HbF synthesis by targeting EKLF in human erythroid cells. Exp Hematol 63:33–40:e32. 10.1016/j.exphem.2018.03.004 10.1016/j.exphem.2018.03.004 29601850

[34] Cheng Y et al (2021) MicroRNA-2355–5p regulates gamma-globin expression in human erythroid cells by inhibiting KLF6. Br J Haematol 193:401–405. 10.1111/bjh.17134 10.1111/bjh.17134 33368182

[35] Ma SP et al (2021) Long noncoding RNA HBBP1 enhances gamma-globin expression through the ETS transcription factor ELK1. Biochem Biophys Res Commun 552:157–163. 10.1016/j.bbrc.2021.03.051 10.1016/j.bbrc.2021.03.051 33744764

[36] Morrison TA et al (2018) A long noncoding RNA from the HBS1L-MYB intergenic region on chr6q23 regulates human fetal hemoglobin expression. Blood Cells Mol Dis 69:1–9. 10.1016/j.bcmd.2017.11.003 10.1016/j.bcmd.2017.11.003 PMC5783741 29227829

[37] Ivaldi MS et al (2018) Fetal gamma-globin genes are regulated by the BGLT3 long noncoding RNA locus. Blood 132:1963–1973. 10.1182/blood-2018-07-862003 10.1182/blood-2018-07-862003 PMC6213316 30150205

[38] Panda AC (2018) Circular RNAs act as miRNA sponges. Adv Exp Med Biol 1087:67–79. 10.1007/978-981-13-1426-1_6 10.1007/978-981-13-1426-1_6 30259358

[39] Syring I et al (2015) Circulating serum miRNA (miR-367–3p, miR-371a-3p, miR-372–3p and miR-373–3p) as biomarkers in patients with testicular germ cell cancer. J Urol 193:331–337. 10.1016/j.juro.2014.07.010 10.1016/j.juro.2014.07.010 25046619

[40] Han F et al (2020) hsa_circ_0001947 suppresses acute myeloid leukemia progression via targeting hsa-miR-329–5p/CREBRF axis. Epigenomics 12:935–953. 10.2217/epi-2019-0352 10.2217/epi-2019-0352 32657138

[41] Hoekstra M et al (2010) The peripheral blood mononuclear cell microRNA signature of coronary artery disease. Biochem Biophys Res Commun 394:792–797. 10.1016/j.bbrc.2010.03.075 10.1016/j.bbrc.2010.03.075 20230787

[42] Langhe R et al (2015) A novel serum microRNA panel to discriminate benign from malignant ovarian disease. Cancer Lett 356:628–636. 10.1016/j.canlet.2014.10.010 10.1016/j.canlet.2014.10.010 25451316

[43] Wang Z, Cao Z (2021) Significance of long non-coding RNA IFNG-AS1 in the progression and clinical prognosis in colon adenocarcinoma. Bioengineered 12:11342–11350. 10.1080/21655979.2021.2003944 10.1080/21655979.2021.2003944 PMC8810008 34872454

