# Supplementary figures and images for: Peripheral blood circular RNA circ-0008102 may serve as a novel clinical biomarker in beta-thalassemia patients
Source: Eur J Pediatr. 2024 Jan 2;183(3):1367–79. doi: 10.1007/s00431-023-05398-y (PMC10950970; doi:10.1007/s00431-023-05398-y)

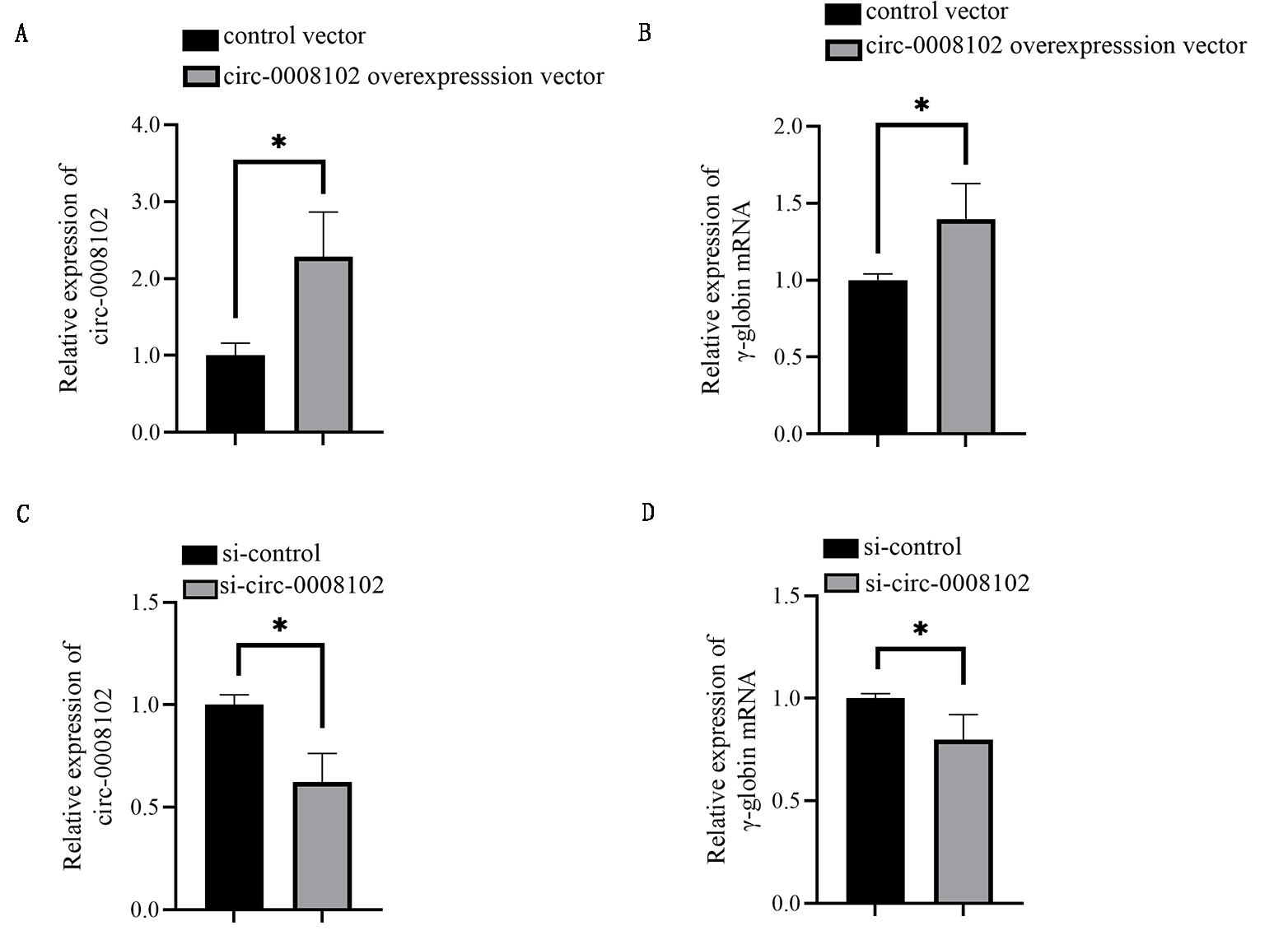

Supplement: Supplementary file 1 — Supplementary file1 (TIF 5258 KB) [file 431_2023_5398_MOESM1_ESM.tif]

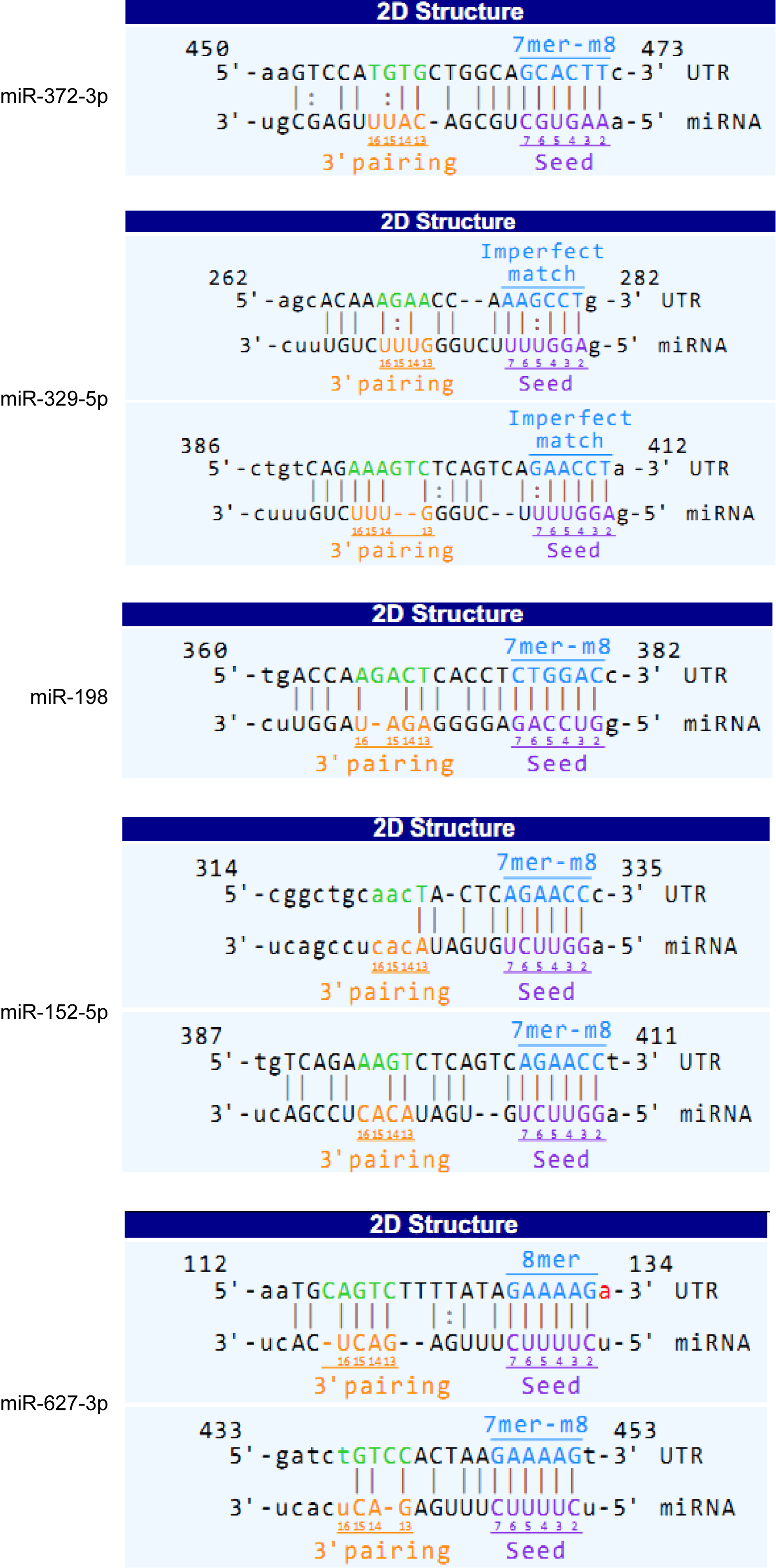

Supplement: Supplementary file 2 — Supplementary file2 (TIF 2021 KB) [file 431_2023_5398_MOESM2_ESM.tif]

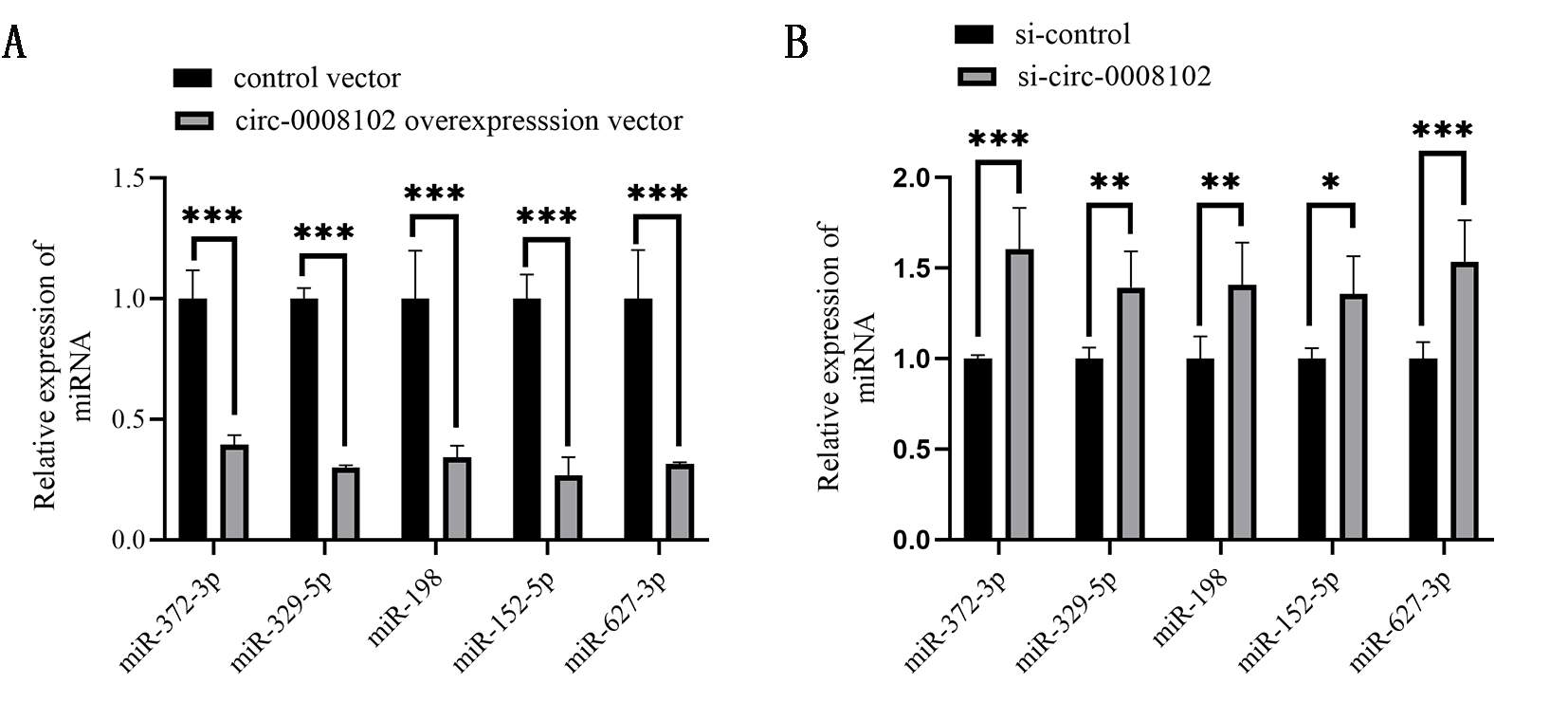

Supplement: Supplementary file 3 — Supplementary file3 (TIF 3610 KB) [file 431_2023_5398_MOESM3_ESM.tif]

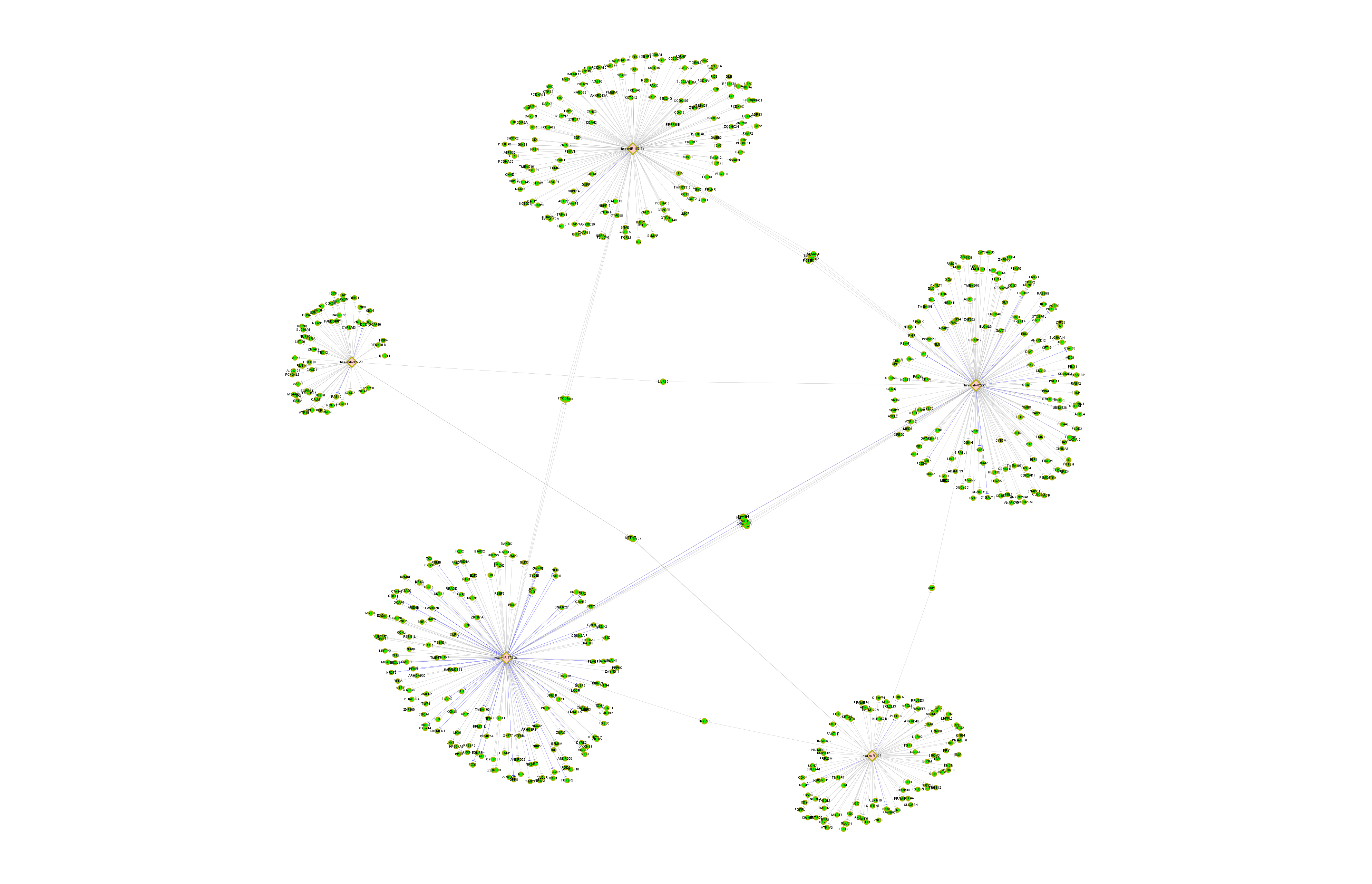

Supplement: Supplementary file 4 — Supplementary file4 (TIF 3397 KB) [file 431_2023_5398_MOESM4_ESM.tif]

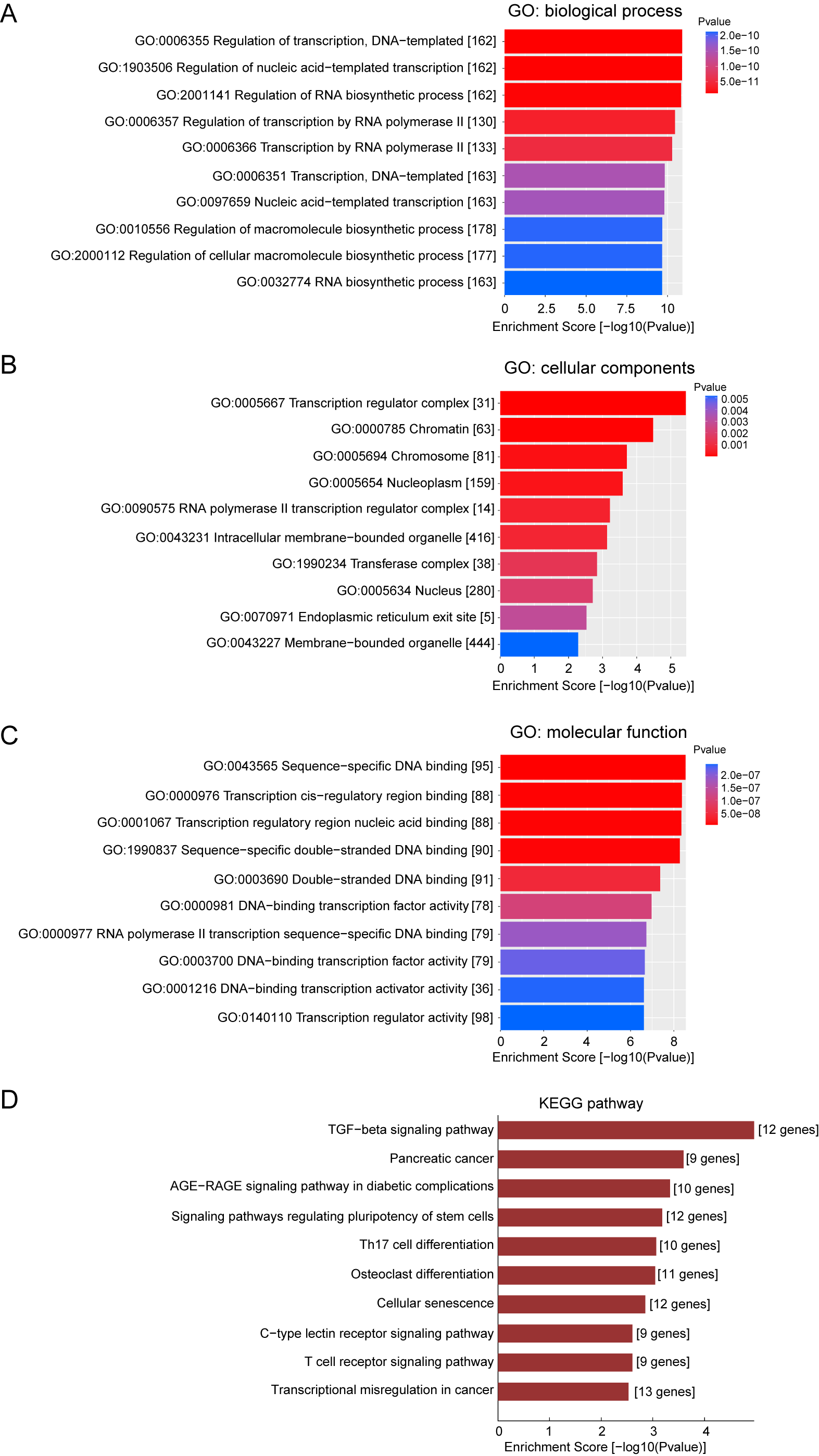

Supplement: Supplementary file 5 — Supplementary file5 (TIF 808 KB) [file 431_2023_5398_MOESM5_ESM.tif]
