# Supplementary material for: Peripheral blood circular RNA circ-0008102 may serve as a novel clinical biomarker in beta-thalassemia patients
Source: Eur J Pediatr. 2024 Jan 2;183(3):1367–79. doi: 10.1007/s00431-023-05398-y (PMC10950970; doi:10.1007/s00431-023-05398-y)
Supplement: Supplementary file 6 — Supplementary file6 (DOCX 14 KB) [file 431_2023_5398_MOESM6_ESM.docx]

**Supplementary Table S1.** The primers of qRT-PCR.

| Gene symbol | Sequences |
| --- | --- |
| circ-0008102 | sense: 5’-GACGGTGTACTTGATCTGTCCA-3’ |
|  | antisense: 5’-GTTGGATCATTCGCTGCATGAT-3’ |
| LCOR | sense: 5’-GACGGACTTCGGAGTGGTGATG-3’ |
|  | antisense: 5’-GAGCCAGTGGAACTTTGAGTGATG-3’ |
| β-globin | sense: 5’-TGTCCACTCCTGATGCTGTTATG-3’ |
|  | antisense: 5’-GGCACCGAGCACTTTCTTG-3’ |
| γ-globin | sense: 5’-CTGGGAAGGCTCCTGGTTG-3’ |
|  | antisense: 5’-CAGAGGCAGAGGACAGGTTG-3’ |
| β-actin | sense: 5’-GCACAGAGCCTCGCCTT-3’ |
|  | antisense: 5’-GTTGTCGACGACGAGCG-3’ |
| miR-372-3p | sense: 5’-AAAGTGCTGCGACATTTGAGCGT-3’ |
|  | antisense: universal primer |
| miR-329-5p | sense: 5’-GAGGTTTTCTGGGTTTCTGTTTC-3’ |
|  | antisense: universal primer |
| miR-198 | sense: 5’-GGTCCAGAGGGGAGATAGGTTC-3’ |
|  | antisense: universal primer |
| miR-152-5p | sense: 5’-AGGTTCTGTGATACACTCCGACT-3’ |
|  | antisense: universal primer |
| miR-627-3p | sense: 5’-TCTTTTCTTTGAGACTCACT-3’ |
|  | antisense: universal primer |
| U6 | sense: 5’-CTCGCTTCGGCAGCACATA-3’ |
|  | antisense: 5’-AACGCTTCACGAATTTGCGT-3’ |

qRT-PCR: quantitative real-time PCR, LCOR: ligand dependent nuclear receptor corepressor. The universal primer provided by Mir-XTM First Strand Synthesis kit.
