# Supplementary material for: Peripheral blood circular RNA circ-0008102 may serve as a novel clinical biomarker in beta-thalassemia patients
Source: Eur J Pediatr. 2024 Jan 2;183(3):1367–79. doi: 10.1007/s00431-023-05398-y (PMC10950970; doi:10.1007/s00431-023-05398-y)
Supplement: Supplementary file 7 — Supplementary file7 (DOCX 20 KB) [file 431_2023_5398_MOESM7_ESM.docx]

**Supplementary Table S2.** The genotypes of pediatric β-thal patients.

| Case no. | Sex | Age (year) | Genotypes |  |
| --- | --- | --- | --- | --- |
| 1 | male | 7 | β^CD41-42(-TCTT)^/β^-28(A>G)^ |  |
| 2 | male | 10 | β^IVS-II-654(C>T)^/β^-28(A>G)^ |  |
| 3 | female | 7 | β^IVS-II-654(C>T)^/β^IVS-II-654(C>T)^ |  |
| 4 | male | 8 | β^CD41-42(-TCTT)^/β^CD26(G>A)^ |  |
| 5 | female | 6 | β^IVS-II-654(C>T)^/β^CD71-72(+A)^ |  |
| 6 | male | 9 | β^CD41-42(-TCTT)^/β^CD41-42(-TCTT)^ |  |
| 7 | male | 9 | β^CD41-42(-TCTT)^/β^CD27-28(+C)^ |  |
| 8 | male | 4 | β^IVS-II-654(C>T)^/β^Int(ATG>AG)^ |  |
| 9 | female | 9 | β^CD41-42(-TCTT)^/β^-28(A>G)^ |  |
| 10 | male | 7 | β^CD41-42(-TCTT)^/β^IVS-I-1(G>T)^ |  |
| 11 | female | 10 | β^CD41-42(-TCTT)^/β^CD41-42(-TCTT)^ |  |
| 12 | male | 7 | β^CD41-42(-TCTT)^/β^CD17(A>T)^ |  |
| 13 | female | 9 | β^CD17(A>T)^/β^CD17(A>T)^ |  |
| 14 | female | 11 | β^CD41-42(-TCTT)^/β^CD17(A>T)^ |  |
| 15 | female | 13 | β^CD41-42(-TCTT)^/β^-28(A>G)^ |  |
| 16 | female | 6 | β^CD41-42(-TCTT)^/β^CD17(A>T)^ |  |
| 17 | male | 10 | β^CD41-42(-TCTT)^/β^-28(A>G)^ |  |
| 18 | male | 5 | β^CD17(A>T)^/β^CD71-72(+A)^ |  |
| 19 | male | 7 | β^CD41-42(-TCTT)^/β^CD71-72(+A)^ |  |
| 20 | male | 9 | β^CD41-42(-TCTT)^/β^CD41-42(-TCTT)^ |  |
| 21 | male | 9 | β^CD17(A>T)^/β^CD17(A>T)^ |  |
| 22 | male | 9 | β^CD17(A>T)^/β^CD17(A>T)^ |  |
| 23 | male | 7 | β^CD41-42(-TCTT)^/β^-28(A>G)^ |  |
| 24 | male | 9 | β^CD41-42(-TCTT)^/β^CD41-42(-TCTT)^ |  |
| 25 | female | 11 | β^CD41-42(-TCTT)^/β^CD41-42(-TCTT)^ |  |
| 26 | male | 5 | β^CD41-42(-TCTT)^/β^CD17(A>T)^ |  |
| 27 | female | 8 | β^IVS-II-654(C>T)^/β^CD17(A>T)^ |  |
| 28 | male | 8 | β^IVS-II-654(C>T)^/β^CD26(G>A)^ |  |
| 29 | male | 11 | β^IVS-II-654(C>T)^/β^CD17(A>T)^ |  |
| 30 | female | 9 | β^CD17(A>T)^/β^CD26(G>A)^ |  |
| 31 | female | 8 | β^CD41-42(-TCTT)^/β^CD17(A>T)^ |  |
| 32 | female | 6 | β^CD41-42(-TCTT)^/β^CD41-42(-TCTT)^ |  |
| 33 | female | 7 | β^CD41-42(-TCTT)^/β^CD41-42(-TCTT)^ |  |
| 34 | female | 10 | β^CD41-42(-TCTT)^/β^IVS-II-654(C>T)^ |  |
| 35 | male | 10 | β^CD41-42(-TCTT)^/β^CD41-42(-TCTT)^ |  |
| 36 | female | 10 | β^CD41-42(-TCTT)^/β^CD17(A>T)^ |  |
| 37 | female | 8 | β^CD17(A>T)^/β^-28(A>G)^ |  |
| 38 | male | 6 | β^CD41-42(-TCTT)^/β^CD41-42(-TCTT)^ |  |
| 39 | female | 5 | β^CD41-42(-TCTT)^/β^CD17(A>T)^ |  |
| 40 | male | 5 | β^CD41-42(-TCTT)^/β^CD17(A>T)^ |  |
| 41 | female | 7 | β^CD17(A>T)^/β^CD17(A>T)^ |  |
| 42 | male | 9 | β^CD41-42(-TCTT)^/β^CD41-42(-TCTT)^ |  |
| 43 | female | 6 | β^CD41-42(-TCTT)^/β^IVS-II-654(C>T)^ |  |
| 44 | male | 6 | β^CD41-42(-TCTT)^/β^CD17(A>T)^ |  |
| 45 | male | 11 | β^CD41-42(-TCTT)^/β^CD17(A>T)^ |  |
| 46 | female | 7 | β^CD17(A>T)^/β^-28(A>G)^ |  |
| 47 | male | 6 | β^IVS-II-654(C>T)^/β^CD26(G>A)^ |  |
| 48 | female | 8 | β^CD41-42(-TCTT)^/β^CD17(A>T)^ |  |
| 49 | male | 7 | β^CD41-42(-TCTT)^/β^CD41-42(-TCTT)^ |  |
| 50 | female | 7 | β^CD41-42(-TCTT)^/β^CD41-42(-TCTT)^ |  |
| 51 | female | 11 | β^-28(A>G)^/β^IVS-I-1(G>T)^ |  |
| 52 | male | 7 | β^CD41-42(-TCTT)^/β^IVS-II-654(C>T)^ |  |
| 53 | male | 8 | β^CD41-42(-TCTT)^/β^CD41-42(-TCTT)^ |  |
| 54 | male | 5 | β^CD17(A>T)^/β^CD26(G>A)^ |  |
| 55 | female | 11 | β^CD41-42(-TCTT)^/β^-28(A>G)^ |  |
| 56 | male | 11 | β^CD41-42(-TCTT)^/β^CD71-72(+A)^ |  |
| 57 | female | 13 | β^CD41-42(-TCTT)^/β^IVS-II-654(C>T)^ |  |
| 58 | female | 10 | β^CD41-42(-TCTT)^/β^CD17(A>T)^ |  |
| 59 | female | 5 | β^CD41-42(-TCTT)^/β^CD41-42(-TCTT)^ |  |

β-thal: β-thalassemia.
