# Supplementary material for: Peripheral blood circular RNA circ-0008102 may serve as a novel clinical biomarker in beta-thalassemia patients
Source: Eur J Pediatr. 2024 Jan 2;183(3):1367–79. doi: 10.1007/s00431-023-05398-y (PMC10950970; doi:10.1007/s00431-023-05398-y)
Supplement: Supplementary file 8 — Supplementary file8 (DOCX 17 KB) [file 431_2023_5398_MOESM8_ESM.docx]

**Supplementary Table S3.** Comparison of clinical characteristics between pediatric β-thal patients without transfusion and pediatric β-thal patients with transfusion.

| Characteristics | Pediatric β-thal patients | | *P* values |
| --- | --- | --- | --- |
|  | without blood transfusion (n=20) | blood transfusion (n=39) |  |
| Age (years) | 7.45±2.24 | 8.46±2.00 | 0.082 |
| Sex (male/female) | 13/7 | 18/21 | 0.176 |
| RBC (×10^12^/L) | 3.42±0.72 | 3.49±0.59 | 0.693 |
| Hb (g/L) | 84.50±18.11 | 95.11±17.37 | 0.034* |
| MCV (fL) | 72.54±15.57 | 84.28±3.09 | <0.001* |
| MCH (pg) | 24.72±1.40 | 27.20±1.29 | <0.001* |
| HbA (%) | 81.44±14.39 | 90.10±8.74 | 0.006* |
| HbA_2_ (%) | 4.12±3.46 | 3.61±2.35 | 0.504 |
| HbF (%) | 14.44±14.98 | 6.29±8.53 | 0.010* |
| BUN (mmol/L) | 5.36±1.72 | 5.20±2.22 | 0.852 |
| Cr (μmol/L) | 28.14±8.50 | 32.48±8.37 | 0.194 |
| UA (μmol/L) | 292.65±135.78 | 286.61±86.82 | 0.887 |
| TP (g/L) | 68.43±5.55 | 68.08±4.74 | 0.843 |
| ALB (g/L) | 43.58±3.76 | 44.54±1.85 | 0.305 |
| TBIL (μmol/L) | 34.84±21.23 | 27.21±10.86 | 0.138 |
| DBIL (μmol/L) | 9.77±4.04 | 9.50±4.52 | 0.858 |
| ALT (U/L) | 56.67±47.63 | 40.65±35.75 | 0.238 |
| AST (U/L) | 52.31±34.92 | 40.90±25.34 | 0.242 |
| GGT (U/L) | 17.26±8.81 | 15.27±8.19 | 0.495 |
| SF (μg/L) | 2331.26±1359.18 | 3090.35±1739.91 | 0.226 |

**P*<0.05 compared to pediatric β-thal patients with transfusion.
