# Supplementary material for: Peripheral blood circular RNA circ-0008102 may serve as a novel clinical biomarker in beta-thalassemia patients
Source: Eur J Pediatr. 2024 Jan 2;183(3):1367–79. doi: 10.1007/s00431-023-05398-y (PMC10950970; doi:10.1007/s00431-023-05398-y)
Supplement: Supplementary file 9 — Supplementary file9 (DOCX 15 KB) [file 431_2023_5398_MOESM9_ESM.docx]

**Supplementary Table S4.** Spearman correlation analysis the correlations between circ-0008102 expression and hematological parameters and biochemical indicators in pediatric β-thal patients without transfusion.

| Parameters | Circ-0008102 expression | | Indicators | Circ-0008102 expression | |
| --- | --- | --- | --- | --- | --- |
|  | Correlation coefficient | *P* values |  | Correlation coefficient | *P* values |
| RBC | 0.521 | 0.026* | BUN | -0.100 | 0.789 |
| Hb | 0.348 | 0.157 | Cr | -0.726 | 0.018* |
| MCV | 0.055 | 0.829 | UA | -0.576 | 0.082 |
| MCH | -0.303 | 0.222 | TP | 0.360 | 0.177 |
| HbA | -0.494 | 0.037* | ALB | 0.445 | 0.170 |
| HbA_2_ | -0.191 | 0.448 | TBIL | -0.224 | 0.484 |
| HbF | 0.480 | 0.044* | DBIL | -0.224 | 0.484 |
| - | - | - | ALT | -0.378 | 0.226 |
| - | - | - | AST | -0.508 | 0.092 |
| - | - | - | GGT | 0.127 | 0.694 |
| - | - | - | SF | -0.267 | 0.488 |

**P*<0.05.
